# Episodic memory, concentrated attention and processing speed in aging: A comparative study of Brazilian age groups

**DOI:** 10.1590/S1980-57642010DN40200003

**Published:** 2010

**Authors:** Rochele Paz Fonseca, Nicolle Zimmermann, Lilian Cristine Scherer, Maria Alice de Mattos Pimenta Parente, Bernadette Ska

**Affiliations:** 1 PhD, Psychology Faculty, Post-Graduate Program in Psychology, Human Cognition, Pontifícia Universidade Católica do Rio Grande do Sul (PUCRS). Coordinator of the Research Group “Neuropsicologia Clínica e Experimental” (GNCE), Porto Alegre RS, Brazil.; 2 Undergraduate Student, Scholarship holder PIBIC-CNPq, Psychology Course, Universidade do Vale do Rio dos Sinos, Pontifícia Universidade Católica do Rio Grande do Sul (PUCRS). Member of the Research Group GNCE (PUCRS).; 3 PhD, Linguistics Faculty, Post-Graduate Program in Linguistics, Pontifícia Universidade Católica do Rio Grande do Sul (PUCRS).; 4 PhD, Psychology Institute, Federal University of Rio Grande do Sul. Coordinator of the Laboratory of Neuropsycholinguistics (Neupsilin), Porto Alegre RS, Brazil.; 5 PhD, Faculté de Médecine, École d’Orthophonie et Audiologie, Université de Montréal, Montréal, Québec, Canada.

**Keywords:** neuropsychological tests, aging, age effect, memory, attention

## Abstract

Neuropsychological studies on the processing of some specific cognitive functions throughout aging are essential for the understanding of human cognitive development from ages 19 to 89.

**Objectives:**

This study aimed to verify the occurrence of differences in the processing of episodic memory, concentrated attention and speed of attentional processing among four age groups of adults.

**Methods:**

A total of 136 neurologically healthy adults, aged 19-89, with 9 or more years of schooling, took part in the study. Participants were divided according to four age groups: young, middle-aged, elderly and oldest old adults. Subtests of the Brief Neuropsychological Evaluation Instrument (NEUPSILIN) were applied for the cognitive assessment. Mean score of corrected answers and of response times were compared between groups by means of a one-way ANOVA test with post-hoc Scheffe procedures and ANCOVA including the co-variables of years of schooling and socio-economical scores.

**Results:**

In general, differences in performance were observed from 60 years old on. Only the episodic memory task of delayed recall reflected differences from the age of around 40 onwards and processing speed from around the age of 70 onwards. Thus, differences were found between the age groups regarding their cognitive performance, particularly between young adults and elderly adults, and young adults and oldest old adults.

**Conclusions:**

Our research indicates that the middle-aged group should be better analyzed and that comparative cross-sectional studies including only extreme groups such as young and elderly adults are not sufficient.

## Abstract (pt)

Os estudos neuropsicológicos sobre o processamento de algumas funções cognitivas específicas no envelhecimento são essenciais para a compreensão do desenvolvimento humano na idade de 19 a 89 anos.

**Objetivos:**

Esse estudo teve como objetivo verificar a ocorrência de diferenças no processamento da memória episódica, atenção concentrada e velocidade de processamento entre quatro grupos etários.

**Métodos:**

Participaram desse estudo 136 adultos neurologicamente saudáveis de 19 a 89 anos, com 9 anos ou mais de escolaridade, divididos em quatro grupos etários: jovens, de idade intermediária, idosos e longevos. Subtestes do Instrumento de Avaliação Neuropsicológica Breve – NEUPSILIN foram utilizados para a avaliação cognitiva. A média dos escores e o tempo de resposta foram comparados entre os grupos através do teste **one-way** ANOVA, com o procedimento post-hoc Scheffe e uma ANCOVA com as co-variáveis anos de escolaridade e escore sócio-econômico.

**Resultados:**

De maneira geral, foram encontradas diferenças entre os grupos a partir dos 60 anos. Somente na tarefa de memória episódica tardia foram encontradas diferenças aproximadamente a partir dos 40 anos, e na velocidade de processamento, a partir dos 70 anos de idade. Dessa forma, houve diferenças entre os grupos no seu desempenho cognitivo, principalmente entre o grupo de adultos jovens e adultos idosos e adultos jovens e adultos longevos.

**Conclusões:**

Os resultados demonstraram, ainda, que o grupo de adultos de idade intermediária deve ser mais bem investigado e, a partir disso, que estudos comparativos incluindo grupos de faixas etárias extremas não são suficientes.

Issues related to aging have been increasingly explored in research on developmental neuropsychology.^1,2^ Neuropsychological aging can be characterized as a heterogeneous process accompanied by compensations which involve both synaptic reorganization and cognitive strategies.^3^ In the literature, no consensus has yet been reached over data regarding cognitive development in healthy aging. Currently, there is a tendency to believe that the preservation and/or the decline of the cognitive functions may be dissociated.^4^ For instance, language ability tends to improve, while working memory and some components of the executive functions tend to decline, particularly after the age of 75 years.^5^

Therefore, one of the main challenges of studies on cognitive aging is the search for a better understanding of the underlying processes which impact functions that heterogeneously change throughout human life span.^6,7^ Moreover, there is a lack of studies in Brazil investigating the effects of sociodemographic variables on adults’ neuropsychological performance using tests constructed and adapted to Brazilian Portuguese. As several previous studies have demonstrated, cognitive performance is influenced by the linguistic characteristics of the multiple ethnic groups, reflecting the need for a better understanding of the differences and similarities in cognitive performance of the various populations.^8^ A greater number of studies seeking to identify and characterize the neuropsychological profile of the Brazilian elderly population is relevant since cognitive processing may be influenced by factors such as gender, educational level, frequency and type of writing and reading habits, as well as number of languages spoken, level of social interaction, cultural aspects among others,^9-14^ all of which are very diverse in the Brazilian population.

Regarding memory, there is a consensus in the literature that this function declines with age. However, a uniform decline of all mnemonic systems does not occur.^15,16^ According to the multiple systems of memory model,^17,18^ episodic memory, or the declarative memory for past events, is one of the most affected functions during aging.^19-21^ On the other hand, semantic memory is relatively spared throughout the aging process.^22^ In this growing field of research, despite the various studies on the effect of different age levels on performance in episodic memory tasks, only a few studies have encompassed all the phases in adult development without having large age gaps between groups.^21,23-25^ In this context, the present study aimed to verify whether differences exist in episodic memory processing, concentrated attention and speed of attentional processing among four groups of adults at different age levels.

## Methods

A total of 136 neurologically healthy adults participated in this study, divided into four age groups:

[1] young adults aged 19-39y (n=52);
[2] middle-aged adults, aged 40-59y (n=26);
[3] elderly adults aged 60-75y (n=24) and oldest old adults aged 76-89y (n=34).

The criteria adopted to organize the groups are in accordance with those used in several psychological and neuropsychological studies.^26,27^ Intervals ranging from 15 to 20 years seem to be ideal for age delimitation of groups in neuropsychology, according to the studies for the normatization of the NEUROPSI^28^ and the Boston Diagnostic Aphasia Examination.^29^ Table 1 presents the participants’ sociodemographic and clinical features.

**Table 1:** Group characterization according to sociodemographic variables.

Variables	Young adults			Middle-aged adults			Elderly adults			Oldest old adults			Total			p*
	M	SD		M	SD		M	SD		M	SD		M	SD		
Age	25.37	6.57		48.19	4.46		66.58	5.41		81	4.32		50.91	23.54		0.001
Gender F/M (n)	36/16	25/1		17/7	25/11		103/33	0.058								
Years of schooling	13.83	2.32		15.42	5.84		14.21	3.24		13.21	3.4		14.05	3.68		0.134
Socioeconomic score (CCEB)^§^	22.19	3.38		22.85	3.54		22.46	3.92		21.35	3.68		22.15	3.58		0.419
Reading and writing habits**	15.9	5.49		15	4.66		14.13	4.87		12.56	5.6		14.58	5.38		0.038
MMSE^||^	+	+		27.83	1.44		28.17	1.87		26.97	1.74		27.57	1.76		0.027
GDS-30^¶^ score	6.6	4.79		5.5	3.84		6.58	5.39		4.68	2.87		5.9	4.36		0.187

* Significance level p≤0.01;

+ Young adults were not evaluated by the Mini-Mental State Exam;

§ Criterion of economic classification in Brazil (http://www.abep.org/codigosguias/ABEP_CCEB.pdf);

|| Mini Mental State Examination (Folstein & Folstein 1975, adapted to the Southern Brazilian population by Chaves & Izquierdo, 1992);

¶ Geriatric Depression Scale (GDS-30) (Yesavage, Brink, Rose, & Lurn, 1983).

** Frequency.

The sample appeared to be homogeneous, that is, the four age groups did not significantly differ in terms of gender distribution (chi-square), years of formal education, socioeconomic score, frequency of reading and writing habits, the Mini-Mental State Examination (MMSE) or Geriatric Depression Scale (GDS-30) scores (one-way ANOVA). The frequency of reading and writing habits was assessed by means of an analysis of the weekly frequency of the habit of reading magazines, newspapers, books and others, and of the habit of writing texts, messages and others. The participants chose from the following response options: everyday (4 points), some days per week (3 points), once a week (2), rarely (1) and never (0). The maximum score for reading habits was 16 points and for writing, 12 points.

The participants were fluent native speakers of Brazilian Portuguese and had completed nine years or more of formal education. Also, they reported no previous or current history of neurological or psychiatric diseases, non-corrected sensory problems or abusive use of cigarettes, alcohol or illicit drugs. Participants medicated with benzodiazepinic drugs during the six months leading up to the evaluation were not included. Problems related to alcohol use were examined using the CAGE Questionnaire (Ewing version used in the study of Amaral and Malbergier^30^) and tobacco addiction by the Fagerström Questionnaire of Tolerance (Fagerström and Schneider version adopted by Marques and cols^31^). In order to reach higher sample homogeneity, all participants belonged to Economic Classes A1, B1 or B2, according to the Brazilian Criterion of Economic Classification CCEB (http://www.abep.org/codigosguias/ABEP_CCEB.pdf). The data described above were assessed by applying a questionnaire on sociocultural data and health aspects.^32^ In order to exclude cases suggestive of moderate or severe depression, the participants had to score less than or equal to 20 on the GDS-30.^33^ The MMSE (Folstein and Folstein version adapted for the Southern Brazilian population by Chaves and Izquierdo^34^) was administered only to adults above 40 years old, and for inclusion in the sample a score greater than or equal to 24 was necessary.

### Instruments

#### Subtests of the Brazilian Brief Neuropsychological Assessment Battery NEUPSILIN^35,36^

***Concentrated Attention – Inverse Counting and Processing Speed Ccore:*** The task requires the participant to count backwards from 50 to 30. Besides the number of correct responses (maximum score of 20 points), the time in seconds is also measured (processing speed).

***Concentrated Attention – Repetition of a Sequence of Digits:*** The participants were asked to pay attention to a numerical sequence and repeat them afterwards in the order they were verbalized by the examiner. The maximum score is seven points.

***Verbal Episodic Memory – Immediate Recall:*** A list of nine words was read to the participants. Immediately afterwards, the examiner asked them to recall the highest number of words possible, not necessarily in the same order of presentation. Participants were told that they would be required to recall the list again later. The maximum score was nine.

***Verbal Episodic Memory – Delayed Recall:*** The Delayed Recall subtest is based on the nine-word list read to the participant in the Immediate Recall task. It is applied after some subtests (15 to 20 minutes), when the tester asks the participant to recall the words they remember from the previous list, accepting words presented at any order. The maximum score is nine.

***Verbal Episodic Memory – Recognition:*** After the Delayed Recall task, the tester reads a list of words and asks the participant to judge whether the words belonged to the word list of the Immediate Recall subtest or not. The maximum score is 18 points.

### Procedures

The participants were invited to join the study via telephone contact or personally at universities, companies or centers with large groups of people. After the free consent form was read and signed, neuropsychological evaluations were performed in silent and individual settings. The project of this study was approved by the Research Ethics Committee of the UFRGS (protocol number 2006530).

### Data analysis

The statistical one-way ANOVA test was used to compare mean scores among groups, with a Scheffe post-hoc procedure (p≤0.01).

## Results

The results of the comparison of mean scores of corrected answers and mean execution times on the backwards counting tasks among the four age groups in the brief neuropsychological evaluation are shown in Table 2.

**Table 2:** Means, standard deviations, and variance analysis of age groups on NEUPSILIN.

Age groupSubtest	Young adults M(SD)*	Middle-aged adults M(SD)*	Elderly adults M(SD)*	Oldest old adults M(SD)*	F	p*	Differences among groups *(post-hoc)*
ABC	19.44 (2.87)	19.85 (0.37)	19.67 (0.82)	19.85 (0.50)	0.46	0.713	None
ABC - T	17.52 (5.16)	19.12 (7.15)	20.76 (7.26)	23.31 (9.99)	4.45	0.005	YA versus OOA
ADR	4.81 (1.97)	3.38 (1.92)	2.75 (2.03)	2.71 (1.57)	11.46	0.001	YA versus EA and OOA
MIR	5.96 (1.61)	4.88 (1.51)	4.42 (1.35)	3.59 (1.16)	19.66	0.001	YA versus EA and OOA; MAA versus OOA
MDR	3.88 (2.45)	2.50 (2.27)	1.88 (1.62)	0.97 (1.09)	15.54	0.001	YA versus EA and OOA
RM	14.37 (2.25)	13.08 (2.48)	12.17 (2.60)	11.21 (2.25)	13.34	0.001	YA versus EA and OOA

* Significance level p≤0.01; YA, young adults; MAA, middle-aged adults; EA, elderly adults; OOA, oldest old adults; ABC, attention-backwards counting; ABC-T, attention backwards counting-time, in seconds; ADR, attention-digits repetition; MIR, memory immediate recall; MDR, memory delayed recall; RM, recognition memory.

The analyses of the data displayed in Table 2 show statistically significant differences in all tasks used in the assessment of attention and of episodic memory, except in the subtest of backwards counting. The post-hoc analysis indicates that the groups which more frequently differentiated were those of young adults versus oldest old adults, as well as young adults versus elderly adults, showing lower scores than the young adults group.

## Discussion

In this comparative study with a developmental perspective, a preliminary observation is that differences were observed by age group in almost all attentional (accuracy and time measures) and mnemonic (accuracy) tasks. The groups whose comparisons more frequently presented differences were young adults and oldest old adults, followed by young adults versus elderly adults. These findings suggest the occurrence of significant differences in cognitive performance in a dissociated manner with a significant performance reduction seen sometimes after the age of around 60 and sometimes after 75 years of age.

Results obtained regarding processing speed are in accordance with previous studies.^15,37-40^ In the present sample analyzed, differences were found between the performance of young adults and oldest old adults, suggesting lower ability at around the age of 75.

No differences were found between groups in relation to concentrated attention, assessed by the inverse counting task. Thus, this finding was not in accordance with the literature, which suggests a decline in this ability with aging.^41-43^ Two hypotheses can be drawn from this finding. The first is that the characteristic of the task may have facilitated elderly adults’ performance. The unlimited task time in this subtest may have benefitted this group since the time pressure variable was not present. The second hypothesis is that the control of the schooling effect may have contributed to a greater homogeneity in performance on this task,^28^ a hypotesis reinforced by the covariance analysis.

In the digit sequence repetition task, young adults significantly differed from elderly and from oldest old adults, mirroring results in the literature, which postulate a decline in span task performance with aging.^37,41,44^ This phenomenon may stem from a reduction in the speed of stimuli processing and a consequent lower capacity of stimuli encoding, or from the greater difficulty in inhibitory control of irrelevant information.^41^ Moreover, a decline in sensory functions may also occur where this would compromise processing of visual and auditory tasks.^45,46^ In the same way, age-related decline may also occur in phonological working memory,^47^ which is an ability required to perform span tasks.

Concerning the episodic memory task of immediate recall, results showed significant differences between the intermediate age group and the oldest old adults group. This partially corroborates the hypothesis which postulates that cognitive decline initiates after maturational processes of the central nervous system are complete.^48^ Cansino^15^ for example, suggests that episodic memory decline initiates during young adulthood at the age of 31. Neurobiological data also corroborate this assumption.^49^ Conversely, Salthouse^48^ postulates the emergence of cognitive decline before 60, but not in all cognitive aspects. Thus, studies should further investigate the onset of episodic memory decline.

Also regarding episodic memory processing in aging and considering the delayed recall task which demanded the maintenance of this memory system for a period of about 20 minutes, significant differences in performance were observed in the comparison among the group of young adults and elderly and oldest old adult groups. It was expected that, due to the greater difficulty imposed by recall latency time and distractor tasks, this task would better discriminate the age groups than the immediate recall task.^21,50,51^ Results demonstrated however that the 20-minute time of latency for word recall did not discriminate age groups any more accurately regarding their performance. This result contributes to the discussion on the temporal duration of short and long memory retention, suggesting that a 20-minute interval is not long enough to distinguish age groups in episodic memory tasks. This may however, represent a possible paradigm to measure medium-term memory. It is possible that a higher latency time of recall would distinguish the young adults’ group from the middle-aged one for instance, as in the study of Malloy-Diniz and cols,^52^ in which elderly and oldest old adults had their performance compared on the Rey Auditory Verbal Learning Test (RAVLT) revealing significant differences in word recall after 30 minutes.

The pattern of performance in immediate and delayed recall was replicated in the recognition task. Young adults performed differently from elderly and oldest old adults, in line with some studies investigating this type of task,^25^ but not corroborating others which found no differences between groups.^16^ Thus, there is not yet consensus in the literature regarding the onset of age-related decline in recognition ability.

Several issues should be further investigated to gain a better understanding of the onset and causes of age-related cognitive declines. For instance, research on aging should start including middle-aged populations in its analyses,^53-55^ since this would contribute to the detection of the initial stages of cognitive decline and lead to a better understanding of its causes.^48,56^ Moreover, longitudinal and cross-sectional studies on aging should consider sociodemographic variables. This is important because multiple factors, including schooling, genetics, personality traits, general health status, reading and writing habits, participation in social activities, among others, are linked to the three possible forms of neuropsychological aging: normal healthy aging, mild cognitive decline and dementia.^55,57^

Concluding, the results reported in this study provided evidence on the changing patterns of performance in the cognitive functions of memory, attentional processing speed and concentrated attention in four age-windows in groups of healthy adults. Further studies should sample several age groups and provide a more detailed and specific exploration of middle-aged groups, an age level that has not been sufficiently studied. Research including only extreme age groups in the adult population may have less value, since it may hinder certain specificities in neuropsychological development throughout adulthood.

Regarding methodological design, longitudinal studies including subjects aged 19 to 90 years of age and/or cross-sectional studies with developmental curves should be conducted that seek to observe and reflect on the strengths and weaknesses of each method.^48,58^ Moreover, neuropsychological investigations developed under paradigms well-established in the literature could help reduce the number of methodological discrepancies found over time. This will enable better interpretation and comparison of published data.^48^
